# The multifaceted benefits of walking for healthy aging: from Blue Zones to molecular mechanisms

**DOI:** 10.1007/s11357-023-00873-8

**Published:** 2023-07-26

**Authors:** Zoltan Ungvari, Vince Fazekas-Pongor, Anna Csiszar, Setor K. Kunutsor

**Affiliations:** 1 Vascular Cognitive Impairment, Neurodegeneration and Healthy Brain Aging Program, Department of Neurosurgery, University of Oklahoma Health Sciences Center, Oklahoma City, OK USA; 2 Oklahoma Center for Geroscience and Healthy Brain Aging, University of Oklahoma Health Sciences Center, Oklahoma City, OK USA; 3 International Training Program in Geroscience, Doctoral School of Basic and Translational Medicine/Department of Public Health, Semmelweis University, Budapest, Hungary; 4 Department of Health Promotion Sciences, College of Public Health, University of Oklahoma Health Sciences Center, Oklahoma City, OK USA; 5 Department of Public Health, Semmelweis University, Budapest, Hungary; 6 International Training Program in Geroscience, Doctoral School of Basic and Translational Medicine/Department of Translational Medicine, Semmelweis University, Budapest, Hungary; 7 Diabetes Research Centre, University of Leicester, Leicester General Hospital, Gwendolen Road, Leicester, LE5 4WP UK

**Keywords:** Cardiovascular disease, Cerebrovascular disease, Blood pressure, Mortality, Healthy aging, Aging, Walking

## Abstract

Physical activity, including walking, has numerous health benefits in older adults, supported by a plethora of observational and interventional studies. Walking decreases the risk or severity of various health outcomes such as cardiovascular and cerebrovascular diseases, type 2 diabetes mellitus, cognitive impairment and dementia, while also improving mental well-being, sleep, and longevity. Dose-response relationships for walking duration and intensity are established for adverse cardiovascular outcomes. Walking’s favorable effects on cardiovascular risk factors are attributed to its impact on circulatory, cardiopulmonary, and immune function. Meeting current physical activity guidelines by walking briskly for 30 min per day for 5 days can reduce the risk of several age-associated diseases. Additionally, low-intensity physical exercise, including walking, exerts anti-aging effects and helps prevent age-related diseases, making it a powerful tool for promoting healthy aging. This is exemplified by the lifestyles of individuals in Blue Zones, regions of the world with the highest concentration of centenarians. Walking and other low-intensity physical activities contribute significantly to the longevity of individuals in these regions, with walking being an integral part of their daily lives. Thus, incorporating walking into daily routines and encouraging walking-based physical activity interventions can be an effective strategy for promoting healthy aging and improving health outcomes in all populations. The goal of this review is to provide an overview of the vast and consistent evidence supporting the health benefits of physical activity, with a specific focus on walking, and to discuss the impact of walking on various health outcomes, including the prevention of age-related diseases. Furthermore, this review will delve into the evidence on the impact of walking and low-intensity physical activity on specific molecular and cellular mechanisms of aging, providing insights into the underlying biological mechanisms through which walking exerts its beneficial anti-aging effects.

## Introduction

The Western world is experiencing a significant demographic shift as the population ages. According to the United Nations, the number of people aged 60 years or older is expected to more than double by 2050, reaching 2.1 billion [1]. With this increase in the aging population comes a greater focus on healthy aging, which involves maintaining physical and mental health as people age [2–4].

One area of research that has gained attention in recent years is the study of determinants of healthy aging in the Blue Zones, regions of the world where people live longer, healthier lives than anywhere else [5–7]. Researchers have identified five Blue Zones around the world, including Okinawa in Japan, Sardinia in Italy, Nicoya in Costa Rica, Icaria in Greece, and the Seventh-day Adventist community in Loma Linda, California. These regions have the highest concentration of centenarians, people who have lived beyond 100 years, and the lifestyles of the people living in these regions have been studied to determine the factors contributing to their longevity and healthy aging. This research has identified several factors that contribute to this phenomenon, including diet, social connectedness, and physical activity [5–7].

One of the key lifestyle characteristics of Blue Zone populations is their high levels of physical activity, which includes regular walking in addition to other low-intensity physical activities. These populations engage in physical activity as part of their daily routine, such as walking to work or for daily errands, gardening, and performing other manual labor activities. Numerous studies have evaluated the evidence linking walking and physical activity in addition to other lifestyle factors in Blue Zones to healthy aging and longevity. In the Nicoya Peninsula of Costa Rica, physical activity is an integral part of daily life. The region’s terrain is hilly, and the residents often walk long distances to work or to visit friends and family. This continuous movement, coupled with a healthy diet rich in whole grains, fruits, and vegetables, contributes to the long and healthy lives of the Nicoyans. Similarly, on the Greek island of Ikaria, where the terrain is rugged, residents engage in extensive walking and often participate in physical labor activities such as farming and goat herding. In Sardinia, walking and other physical activities also play a crucial role in healthy aging.

To promote healthy aging, health promotion programs should focus on the determinants of healthy aging identified in the Blue Zones. Regular physical activity, including walking, is a fundamental aspect of a healthy lifestyle and is associated with numerous health benefits, particularly in the context of healthy aging and longevity in the Blue Zones. Therefore, health promotion programs designed to promote healthy aging should prominently include recommendations for walking in addition to other forms of regular physical activity, as a way to improve overall health and well-being.

Though the terms “physical activity” and “exercise” are commonly used interchangeably, they are not necessarily the same. Physical activity is defined as any bodily movement produced by skeletal muscles that requires energy expenditure and includes exercise as well as usual occupational and/or domestic activity [8]. In contrast, exercise is intentional physical activity and can include aerobic training, high-intensity interval training, or resistance training [9]. The evidence supporting the health benefits of physical activity and exercise training is extensive and consistent. Regular physical activity is linked to a reduced risk or severity of adverse vascular outcomes, such as cardiovascular disease (CVD) and type 2 diabetes (T2D), as well as non-vascular outcomes, including various cancers, osteoarthritis, and infectious diseases [10–12]. Importantly, the beneficial health effects of physical activity are irrespective of age, sex, ethnicity, or the presence of comorbidities [13]. Furthermore, regular physical activity and exercise training are also well documented to increase levels of cardiorespiratory fitness (CRF) [14, 15], which is one of the strongest predictors of adverse cardiovascular outcomes [16–18]. Cardiorespiratory fitness is often characterized as maximal oxygen uptake (VO_2max_) or peak VO_2_ (VO_2peak_).

Promoting physical activity has been an important strategy to specifically reduce the prevalence and incidence of common cardiometabolic conditions all over the world. Components of physical activity include frequency, duration, and intensity, which together comprise the volume. To derive the maximal benefits of physical activity, an appropriate intensity, frequency, and duration are required.

Physical activity can also be classified based on the level of intensity: light, moderate, and vigorous. Despite current physical activity guideline recommendations, which state that adults should engage in at least 150–300 min of moderate-intensity physical activity or 75–150 min of vigorous-intensity physical activity per week or an equivalent combination of both types of physical activity per week [8, 9, 19], there is a research gap regarding the dose-response association between volume and intensity of physical activity and health outcomes [8, 9].

Walking is the most commonly reported physical activity and is often classified as light or brisk. Light walking is classified as low-intensity physical activity and brisk walking as moderate intensity physical activity. While the cardiovascular benefits of walking are widely acknowledged, there is uncertainty regarding the ideal “dose” required to reap cardioprotective benefits, as well as the impact of walking on non-vascular outcomes. Conflicting data also suggests that the intensity of physical activity may be associated with greater benefits than the quantity [20, 21].

The objective of this review is to provide a comprehensive summary of the extensive literature on the health benefits of walking in older adults, including the cardiovascular benefits and postulated biologic mechanisms underlying the associations between walking and health outcomes. Additionally, this review aims to examine the implications for clinical practice and population health and to provide recommendations for future research directions. Specifically, this review will explore the role of walking in promoting healthy aging and improving health outcomes in older adults, with a focus on the specific recommendations that should be included in health promotion programs targeting physical activity, particularly walking. Furthermore, the review will investigate the anti-aging effects of walking, offering valuable insights into the potential contribution of walking to healthy aging.

## Health benefits of walking

### Methods

We conducted a thorough search for observational studies, including prospective cohort, nested case-control, case-cohort or retrospective cohort studies, randomized controlled trials (RCTs), and non-RCTs from MEDLINE and EMBASE up to May 2023. Our search focused on the cardiovascular benefits of walking, with a particular emphasis on robust systematic reviews and meta-analyses of these study designs when available, according to the hierarchy of evidence [22]. Our search terms included a range of keywords related to “walking” and cardiovascular health, such as “cardiovascular disease,” “coronary heart disease,” “sudden cardiac death,” “heart failure,” “hypertension,” and “blood pressure,” as well as keywords related to other health outcomes, including “dementia,” “depression,” “anxiety,” “pulmonary disease,” “sleep,” “fracture,” “mortality,” “lipids,” “inflammation,” “oxidative stress,” “arterial stiffness,” “arterial compliance,” and “intima media thickness.” We restricted our review to studies conducted in human populations, reported in English, and in adults.

While “steps per day” is a commonly used metric for quantifying physical activity, it captures all types of activities involving “a movement made by lifting your foot and putting it down in a different place,” including walking and running [23]. As such, this review did not specifically focus on studies that used the measure of “steps per day.” However, we included studies that captured walking activities using accelerometer-derived daily step count, such as 40 steps/min or faster defined as intentional walking or purposeful movement or 100 steps/min defined as moderate walking pace or brisk walk [24]. We excluded studies that examined walking in combination with other physical activity types such as cycling. Additionally, cross-sectional studies were not included as they do not address temporality. An important goal of this review was to provide a comprehensive overview of the existing evidence on the cardiovascular and other health benefits of walking and to explore the biologic mechanisms underlying these associations in promoting healthy aging.

### Cardiovascular outcomes

#### Cardiovascular risk factors

Several observational cohort and interventional studies have explored the impact of walking on cardiovascular risk factors. However, the results of these individual studies have been conflicting, leading to several systematic reviews and meta-analyses on the topic. One meta-analysis conducted by Kelley and colleagues [25] evaluated the effects of walking on resting systolic blood pressure (SBP) and diastolic blood pressure (DBP) by pooling data from 16 RCTs and non-RCTs. The study showed that walking exercise programs led to mean reductions in SBP and DBP of 3 and 2 mmHg, respectively. Another meta-analysis by Murphy and colleagues [26] pooled data from 24 RCTs to quantify the effect of walking interventions on selected risk factors, including aerobic fitness, blood pressure, and measures of body composition. The study showed that walking interventions increased aerobic fitness and decreased body weight, body mass index (BMI), percent body fat, and resting DBP in sedentary adults. Other meta-analyses have shown that walking significantly decreased glycated hemoglobin (A1c), BMI, and DBP, and increased VO_2max_, while having no effect on high-density lipoprotein cholesterol (HDL-C) or low-density lipoprotein cholesterol (LDL-C) levels [27–32]. Overall, these findings suggest that walking is associated with significant improvements in cardiovascular risk factors and also has the potential to be used as a therapeutic tool for individuals with T2D [33].

Regular exercise, including walking, has a profound impact on endothelial function, which plays a critical role in cardiovascular health [34–38]. Endothelial cells line the inner surface of blood vessels and are responsible for regulating vascular tone and thereby blood pressure, maintaining vascular integrity, regulating hemostasis, and platelet aggregation. The preservation of endothelial health is paramount in preventing the development of atherosclerosis and pathological vascular remodeling in large vessels. Equally important is the maintenance of microvascular endothelial health, as it plays a crucial role in preserving capillary architecture, regulating the tone of resistance arteries to ensure adequate nutrient and oxygen delivery to tissues, and maintaining barrier function, such as the integrity of the blood-brain barrier. Additionally, microvascular endothelial cells are involved in modulating the exchange of molecules, regulation of immune cell function, including leukocyte extravasation, and supporting the maintenance of stem cell niches. Aging is commonly associated with generalized endothelial dysfunction, which negatively impacts the proper functioning of both the large vessels and the microcirculation [39–42]. This age-related endothelial dysfunction compromises the overall health and disrupts the homeostasis of various tissues and organ systems. Age-related endothelial dysfunction contributes to the development of macrovascular (atherosclerotic diseases, including stroke, coronary artery disease, peripheral artery disease) and microvascular diseases (including microvascular pathologies affecting the heart brain, kidneys, skeletal muscle) [39–42]. Exercise promotes endothelial health in aging by promoting an anti-inflammatory, anti-atherogenic gene expression profile, stimulating the production and release of vasodilator nitric oxide (NO), and promoting angiogenesis [43–46]. Exercise-induced shear stress plays a crucial role in regulating various aspects of endothelial function and phenotype. Increased blood flow and shear stress during exercise trigger the release of endothelial-derived NO, leading to vasodilation, lowering blood pressure, and improving tissue perfusion [45–48]. This vasodilatory effect not only enhances oxygen and nutrient delivery, but also facilitates the removal of waste products. Shear stress can modulate the expression of pro-atherogenic and anti-atherogenic genes and activates various intracellular signaling pathways, including those involved in antioxidant defense and vascular remodeling. These mechanisms collectively contribute to the maintenance of a youthful endothelial phenotype and the prevention of endothelial dysfunction and atherosclerotic plaque formation. By promoting favorable shear stress and regulating gene expression, exercise serves as a powerful modulator of endothelial function. By enhancing endothelial function, exercise promotes optimal cardiovascular function and vascular health in older adults and reduces the risk of endothelial dysfunction-associated conditions such as hypertension, atherosclerosis, and CVD.

#### Hypertension

The blood pressure–lowering effect of walking has been widely investigated in observational cohort studies [27, 31, 32, 49, 50] and RCTs, as discussed in the previous section on cardiovascular risk factors. Prospective cohort studies have also reported associations between walking and the risk of hypertension. For instance, a study of 6017 Japanese men (aged 35–60 years) without a history of hypertension or diabetes at baseline found that walking for longer durations was associated with a reduced risk of hypertension [51]. Specifically, compared to a walk of 10 min or less, an 11- to 20-min walk and a walk of 21 min or more were associated with a 12% and 29% lower risk of hypertension, respectively [51]. Similarly, a study of 15,357 university graduates initially free of chronic disease or hypertension found that a normal, brisk, or very brisk walking pace was each associated with a reduced risk of hypertension compared to a slow walking pace, after adjusting for established risk factors [52]. Moreover, a recent prospective analysis of 83,435 postmenopausal women (aged 50–79 years) without known hypertension, heart failure, coronary heart disease (CHD), or stroke found that walking at guideline-recommended volumes (>7.5 MET hours per week) and at faster speeds (≥2 miles per hour) was associated with a lower risk of hypertension [53].

#### Cardiovascular and cerebrovascular diseases

Several prospective epidemiological studies have been conducted to investigate the associations between walking and CVD outcomes [54]. In 2008, Hamer and Chida conducted the first systematic review and meta-analysis of these studies [55], which included 18 prospective studies comprising 459,833 participants free from CVD at baseline with 19,249 CVD cases at follow-up. The authors found that comparing the highest versus the lowest walking category was associated with a 31% reduced risk of CVD. Another meta-analysis by Zheng and colleagues in 2009 found that an increase of approximately 30 minutes of normal walking a day for 5 days a week was associated with a 19% reduction in CHD risk, with no evidence of a difference between men and women [56]. Other studies have reported dose-response reductions in CVD risk with higher walking duration, distance, energy expenditure, and pace [57, 58]. Additionally, walking at a brisk/fast pace was associated with a 24% and 21% reduced risk of CVD mortality, respectively, compared with walking at a slow pace [59]. Other studies reached similar conclusions. In analysis of the UK Biobank comprising 318,185 participants, Celis-Morales and colleagues [54] investigated the associations between usual walking pace and a range of health outcomes. In fully adjusted models, compared to slow pace walkers, men and women with a brisk walking pace had a 38% and 53% reduced risk of CVD mortality, respectively [54]. In another analysis of the UK Biobank cohort, slow walking pace compared with average walking pace was associated with a higher risk of stroke (hazard ratio, HR = 1.45) in the overall study population of 363,137 participants [60]. In a subgroup analysis, the association was only existent among participants aged ≥65 years (HR = 1.42) [60]. In pooled analysis of 8 prospective cohort studies that examined the association between walking pace and stroke risk, individuals in the fastest walking-pace category had a 44% lower risk of stroke compared to individuals in the slowest walking-pace category [61]. In dose-response analysis, every 1 km/h increment in baseline walking pace was associated with a 13% decreased risk of stroke [61]. In addition to walking pace (intensity), a number of individual studies have reported incremental dose-response reductions in the risk of adverse cardiovascular outcomes in relation to increasing walking duration or distance and higher energy expenditure from walking [62–68]. In a recent analysis of the UK Biobank in which data on accelerometer-measured daily step count was available for 78,500 individuals, more daily steps (including purposeful or intentional walking steps) were associated with lower CVD incidence and mortality [69].

### Cognitive function and dementia

The evidence regarding the relationship between physical activity and adverse cognitive outcomes such as cognitive impairment, Alzheimer’s disease (AD), and dementia has been inconsistent. While some studies have reported decreased dementia risk with higher physical activity [70, 71], others have found no association [72–74]. Similar inconsistencies have been found in individual studies of walking with cognitive outcomes. However, a meta-analysis of 17 prospective cohort studies evaluating the association of walking pace with the risk of cognitive decline and dementia among elderly populations found that comparing the lowest to the highest category of walking pace was associated with an increased risk of cognitive decline (relative risk, RR = 1.89) and dementia (RR = 1.66) [75]. Moreover, with every 1 dm/s (360 m/h) decrement in walking pace, the risk of dementia increased by 13% [75]. Another study assessed the dose-response association of daily step count and intensity with the incidence of all-cause dementia among adults [76]. It found that approximately 9800 steps per day may be optimal to reduce the risk of dementia; a minimum dose of 3800 steps per day was associated with a 25% lower risk of dementia [76]. In addition, steps performed at higher intensity resulted in stronger associations [76]. While the evidence is not yet definitive, these studies suggest that walking and higher levels of physical activity may be beneficial for cognitive health. There is increasing evidence that microvascular pathologies play a critical role in the pathogenesis of cognitive impairment and dementia [77–84]. While the exact mechanisms are still being studied, it is becoming increasingly clear that walking and other forms of physical activity have a more profound effect on endothelial cell physiology and the genesis of vascular cognitive impairment (VCI) than on amyloid pathologies within the brain parenchyma. As such, it may be important for future studies to separately investigate the effects of walking on VCI and AD, in order to better understand the complex relationships between physical activity, brain health, and cognitive outcomes.

### Type 2 diabetes mellitus

In a 2007 meta-analysis of 10 prospective studies that investigated the association between moderate-intensity physical activity and the risk of T2D, the pooled analysis of 5 studies specifically evaluating the role of walking showed that regular walking (approximately ≥2.5 h/week) was associated with a 30% reduced risk of T2D compared with almost no walking [85]. In 2020, Ballin and colleagues [86] examined the association between daily step count (assessed with accelerometer with activity threshold set to >100 counts/min) and incident diabetes in 3055 community-dwelling 70 year olds. Participants who took ≥ 4500 steps/day had a 59% lower risk of diabetes compared to those taking fewer steps. Furthermore, the dose-response analysis indicated a steep decline in the risk of diabetes until around 6000 steps/day, with the risk decreasing at a slower rate until it levelled off at around 8000 steps/day [86]. In a recent analysis of 162,155 UK Biobank participants, both average and slow walking pace were each associated with a higher risk of incident T2D compared to brisk walking in both men and women, independent of major confounding factors [87]. Furthermore, recent results from the population-based prospective cohort Hispanic Community Health Study/Study of Latinos, which included 6634 adults, demonstrated that accumulating more daily steps (including purposeful walking steps or brisk walk) and greater step intensity were associated with a reduced risk of diabetes [88].

### All-cause mortality

In the study by Hamer and Chida [55], which investigated the relationship between walking and the risk of all-cause mortality, the highest versus the lowest walking category was associated with a 32% reduced risk of mortality. Similar to the findings for CVD, the results were not significantly different for men and women, with walking pace being a stronger independent predictor compared to walking volume [55]. High walking volume or intensity was associated with the strongest risk reduction [55]. In a pooled analysis of 14 prospective cohort studies, Kelly and colleagues [50] showed an incremental reduction in the risk of all-cause mortality with high walking volume, with a standardized dose of 11.25 MET-hours per week being associated with an 11% risk reduction.

Stamatakis and colleagues found that walking at an average or brisk/fast pace was associated with a 20% and 24% reduced risk of all-cause mortality, respectively, compared to walking at a slow pace [59]. In an analysis of the UK Biobank cohort, Celis-Morales and colleagues found that men and women with a brisk walking pace had a 21% and 27% reduced risk of all-cause mortality, respectively, compared to slow pace walkers [54]. Furthermore, more daily steps, including purposeful or intentional walking steps, up to approximately 10,000 steps, were associated with a lower risk of all-cause mortality in the UK Biobank analysis [69]. A study of 17,466 women (aged 62–101 years) found that approximately 4400 steps per day was associated with a 41% reduction in mortality rate compared with approximately 2700 steps per day, with a steady decline in mortality rates up to approximately 7500 steps per day, beyond which mortality rates levelled [89]. However, the time spent at a stepping rate of 40 steps/min or faster (intentional walking) was not clearly related to mortality risk.

In a recent meta-analysis of 15 international cohorts investigating the associations of daily step count and stepping intensity with all-cause mortality, it was demonstrated that taking more steps per day was associated with progressively lower risk, up to a level that varied by age: 6000–8000 steps per day among adults aged 60 years and older and 8000-10,000 steps per day among adults younger than 60 years [24]. However, the time spent walking at 40 steps/min or faster (intentional walking) and 100 steps/min or faster (defined as moderate rate walking pace) was not found to be significantly associated with mortality [24].

### Cancer

Stamatakis and colleagues [59] conducted a prospective pooled analysis of 11 population-based baseline surveys in England and Scotland in 2018. Their findings reported no evidence of an association between walking pace and cancer mortality. Similarly, Celis-Morales and colleagues [54] found no evidence of associations between walking pace and all-cause cancer, colorectal, and breast cancer in their analysis of the UK Biobank cohort; however, brisk walking was associated with a higher risk of prostate cancer. On the other hand, a recent analysis of the UK Biobank cohort, which measured accelerometer-based daily step count in 78,500 individuals, showed that accruing more daily steps, including intentional walking steps, was associated with a lower risk of incident cancer and mortality due to cancer [69].

### Respiratory pathologies

Celis-Morales and colleagues [54] found, in their analysis of the UK Biobank cohort, that brisk walking was associated with reduced risk of respiratory disease in both men and women. Compared to slow pace walkers, men and women with a brisk walking pace had a 34% lower risk of respiratory disease. The corresponding risk reduction for chronic obstructive pulmonary disease was even greater, at 65% and 72%, respectively. Furthermore, several prospective studies have reported that daily walking habits are associated with a reduced risk of pneumonia-related mortality in older people, with risk reductions ranging from 10 to 42% [90–92]. Although two of the studies did not consider other forms of physical activity besides walking, one study demonstrated that daily walking alone was sufficient to reduce pneumonia-related mortality among older people who do not engage in other exercise habits regularly [90].

### Bone health

Regular physical activity and exercise have been shown to have a positive impact on bone health, reducing the rate of bone loss, conserving bone tissue, increasing bone mineral density (BMD), and lowering the risk of fractures [93, 94]. Weight-bearing endurance activities, muscle-strengthening physical activity, balance exercise, and resistance exercise are recommended in various guidelines to preserve bone health and reduce the risk of falls [94, 95]. However, there is uncertainty about the type and intensity of exercise that is beneficial for bone health. Although some systematic reviews and meta-analyses of RCTs have shown no significant effect of regular walking on BMD in perimenopausal and postmenopausal women [96, 97], others have demonstrated a positive effect on lumbar BMD but not on the femur or calcaneus [98]. One conclusion is that walking alone is not sufficient for those at risk of osteoporosis, and that other forms of exercise in addition to walking should be incorporated [98]. Nevertheless, a recent study suggested that a training program comprising fast walking and running exercises may increase or preserve BMD at the femoral neck in postmenopausal women [99]. Pooled analysis of results from RCTs and quasi-RCTs of adults with chronic musculoskeletal pain showed that walking was associated with significant improvements in pain and function, but the longer-term effectiveness was uncertain [100]. Analysis of data from the Nurses’ Health Study and the Women’s Health Initiative prospective cohort study showed that walking was associated with a lower risk of hip fracture among postmenopausal women [101, 102]. However, in a 5-year follow-up of an Australian population-based prospective study comprising postmenopausal women and men aged 50 years or older, individuals who walked more than 3 h per week had an increased risk of fractures compared with those who reported no walking [103]. Overall, while walking may have some positive effects on bone health, it is important to consider incorporating other types of exercise to optimize bone health outcomes.

### Sleep health

Regular physical activity has been shown to improve sleep quality and duration, but there is ongoing debate regarding the types of physical activity that are most effective in promoting better sleep [104, 105]. Wilbur et al. conducted a RCT to evaluate the impact of a 24-week, home-based, moderate-intensity walking intervention on various menopausal symptoms, including sleep, in 173 sedentary midlife women (aged 45–65 years) [106]. The study found that the frequency of adherence to walking significantly influenced a positive change in sleep symptoms. In a longitudinal study of 103 midlife women (average age = 53, range 40–60 years), increased activity levels during the day were associated with an increase in total sleep time at night, with a stronger protective effect observed in overweight and obese women [106]. A recent 4-week RCT that assessed the effect of walking on sleep quality and duration in 59 healthy participants (average age of 49 years) observed a positive relationship between daily active minutes and sleep quality, but not duration. Women who were more active and took more steps also reported better sleep quality compared to those who were less active [107].

### Mental health conditions and quality of life

Physical activity and exercise have well-documented mental health benefits, even at levels below public health recommendations [108–111]. Studies have shown that physical activity is associated with a reduced risk of depression, with evidence suggesting a causal relationship [112]. In addition to the physical health benefits of walking, it has the potential to enhance emotional and psychological well-being, improve mood, and reduce the risk of various mental conditions. Recent research has demonstrated the effectiveness of walking in reducing the symptoms of depression compared to non-walking interventions such as social support, stretching, and cognitive interventions [113]. Sessions ranged from 20 to 50 min per day to 5 times per week over 6.2 days to 6 months [113]. In another study, brisk walking was found to improve mood state [114], and walking has also been shown to boost creative inspiration. People’s creative output increased by an average of 60% while walking compared to sitting, according to experiments by Oppezzo and Schwartz [115]. Furthermore, walking has been positively linked to various aspects of health-related quality of life [116, 117].

## Cellular and molecular pathways contributing to the anti-aging health benefits of low-intensity exercise and walking

### Endocrine and metabolic pathways

**Fig. 1:**
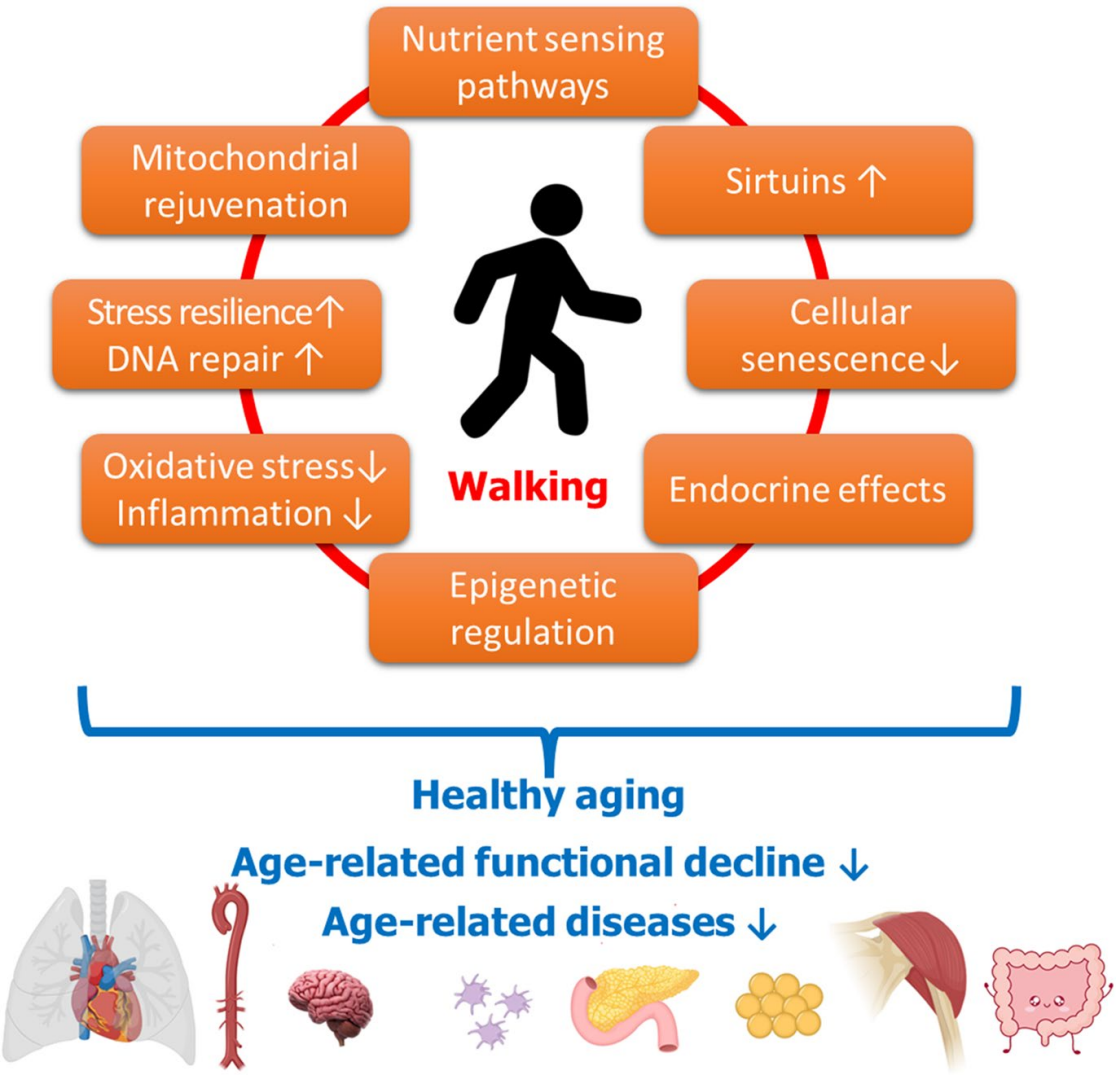
Mechanisms mediating the anti-aging health benefits of light physical exercise and walking. This figure illustrates how light physical exercise and walking contribute to healthy organismal aging by potentially reversing or attenuating underlying cellular and molecular mechanisms of aging. By preventing or delaying age-related functional decline and the onset of age-related diseases in multiple organ systems, exercise and walking promote overall anti-aging effects

The health benefits of physical activity or exercise training are well documented and observed across multiple organ systems including the cardiovascular system. These benefits are achieved through several mechanisms, such as improvements in intermediate or cardiovascular risk factors including BMI, blood pressure, endothelial function, blood glucose, and insulin resistance [27, 29] (Fig. 1). This is consistent with some studies of the associations between walking and adverse cardiovascular outcomes which have reported incomplete attenuations of the associations following adjustment for cardiovascular risk factors such as BMI [58]. However, evidence suggests that these pathways may not completely account for the effects of physical activity or exercise on cardiometabolic health. Emerging evidence indicates that exercise triggers the release of exerkines, which exert their effects through endocrine, paracrine, and/or autocrine pathways [118]. Exerkines have potential roles in improving cardiovascular, metabolic, immune, and neurological health. For instance, exerkines produced by the cardiovascular system could mitigate systemic inflammation and ischemia, while those produced in adipose tissue enhance lipolysis, thermogenesis, and glucose metabolism. Extracellular vesicles have also been implicated in mediating the systemic benefits, including anti-aging effects, of exercise [119–128]. These vesicles encompass all membranous structures that cells secrete and were proposed as mediators of intercellular communication in both physiological and pathological conditions [129–131]. Although their exact function is not yet well understood, they may modulate immune responses, metabolism, angiogenesis, tissue maintenance, and repair [119–128, 132] through cell non-autonomous mechanisms. The observation that senescent cells exhibit an increased release of extracellular vesicles, coupled with an altered compositional profile, posits a compelling implication in their role as mediators of paracrine senescence during the aging process [133–138]. During exercise, muscle and other tissues increase the release of extracellular vesicles with a cargo that may contribute to the mediation of systemic effects of exercise [119–128, 139]. Physical activity is known to have favorable effects on lipid metabolism, reducing levels of serum triglycerides and LDL-C and increasing levels of HDL-C. It has been reported that 3.5–7 h of moderate to vigorous physical activity per week or 30–60 min of exercise on most days could reduce triglycerides by up to 50%, reduce LDL-C by up to 5% and increase HDL-C by 5–10% [13]. However, the evidence collected so far suggests that walking may not have significant effects on lipid profiles [27, 29]. This observation may be related to the intensity of physical activity, as walking may not provide enough intensity to improve lipid profiles, especially in those with hypercholesterolemia or hypertriglyceridemia [13]. The cardiovascular benefits of walking may also be influenced by confounding or interaction with other physical activity types, given that those who walk may also engage in other types of physical activity that have a protective effect on cardiovascular risk [140]. Furthermore, walking is an enjoyable physical activity that can reduce stress, enhance psychological well-being and trigger the release of endorphins, which promote relaxation and improve mood [141]. It is well known that ongoing stress is associated with an increased risk of physical ailments including CVD and cancer, as well as mental health issues and adverse effects on overall health [142].

Physical activity plays a crucial role in influencing hormonal changes associated with aging, particularly in relation to insulin-like growth factor 1 (IGF-1) [143–146]. Epidemiological studies have consistently shown that circulating IGF-1 levels decline with age, which has been linked to the development of various age-related conditions and diseases [147–150]. Interestingly, centenarians have been found to have higher levels of IGF-1 compared to younger individuals [151]. Animal studies further support the pleiotropic anti-aging effects of IGF-1 [151, 152], including its atheroprotective, microvascular protective, and neuroprotective properties [79, 151, 153–160]. While genetic IGF-1 deficiency in humans is associated with shortened lifespan [161], certain genetically modified mouse models with IGF-1 deficiency show lifespan extension due to its anti-cancer growth action [162]. In preclinical models of aging, exercise has been shown to enhance skeletal muscle mass, exercise capacity, metabolism indicators, and protein synthesis, while reducing oxidative stress and apoptosis through the activation of the IGF-1 pathway [146]. However, studies on the effect of physical activity on IGF-1 levels in humans have yielded mixed results [143, 145, 163–166], with some suggesting that physical activity increases circulating IGF-1 levels [143, 167]. The specific effects of walking on IGF-1 levels are less well understood and require further investigation [167, 168].

Age-related changes in sex hormone levels, such as testosterone and estrogen, are also well-documented phenomena of aging [169]. In men, declining levels of androgens, including testosterone, are associated with decreased muscle mass and strength, reduced bone mineralization, and increased central body fat [169]. Resistance training has been shown to increase testosterone levels [169]. As for walking, studies suggest that individuals who take more than 4000 steps daily are less likely to have low testosterone levels, with an approximate increase of 7 ng/dL for every additional 1000 steps taken [170].

In women, estrogen levels decrease with age, which can impact longevity [169]. Estrogen plays a role in antioxidant activity, membrane stabilization, and maintenance of bone mass [169]. Physical activity, including exercise programs, can help counterbalance the decline in estrogen levels observed in postmenopausal women [169]. A 12-week exercise program, for example, has been shown to improve estradiol levels in postmenopausal women, with anaerobic exercises potentially having a more substantial effect compared to aerobic exercises [171]. Further research is needed to investigate the specific effects of walking interventions on estrogen levels in older females.

Understanding the intricate relationship between physical activity, hormonal changes, and the aging process is essential for developing comprehensive interventions to promote healthy aging and prevent age-related diseases. Further studies are warranted to explore the mechanisms and differential effects of walking interventions on hormone levels in older adults, with the ultimate goal of optimizing health outcomes and improving overall well-being.

While the health benefits of physical activity and nutrition are often studied separately, it is widely recognized that they are both vital aspects of a healthy lifestyle and contribute to healthy aging. Integrating nutrition and physical activity can yield more substantial positive health outcomes compared to approaches that solely focus on one or the other [172]. This comprehensive approach recognizes that optimal nutrition plays a crucial role in facilitating exercise performance and enhancing the beneficial effects of exercise. Incorporating natural food components with physiological actions, often referred to as “functional foods” [173, 174], can provide essential nutrients, improve performance and endurance, enhance muscle strength, prevent injury and fatigue, and maintain immunity [175]. Moreover, nutrition therapy has emerged as a promising approach to increasing cardiorespiratory fitness levels among diverse populations with exercise limitations, including those with chronic obstructive pulmonary disease, heart failure, obesity, sarcopenia, and frailty [14, 176–181]. By combining physical activity and optimal nutrition, individuals can optimize their overall health, promote healthy aging, and enhance their quality of life [182–191].

### Effects on cellular and molecular mechanisms of aging

In this section, we explore the effects of low intensity exercise on the fundamental molecular and cellular mechanisms of aging, drawing primarily from experimental studies conducted on rodent models with different exercise paradigms in laboratory settings. Wherever available, we will also discuss relevant human data on the effects of exercise on these mechanisms. These studies have shed light on potential mechanisms through which exercise may exert its beneficial effects on aging. However, it is important to acknowledge that while we review these mechanisms for the benefit of the reader, the direct extrapolation of findings from rodent studies to the effects of walking in humans is still speculative and warrants further investigation. Additionally, extrapolating results on the effects of different exercise regimens from human studies to the specific context of walking can also be challenging, given the unique characteristics and physiological responses associated with this specific mode of physical activity.

The current view is that, in general, exercise exerts multifaceted effects on synergistic cellular and molecular mechanisms that underlie the aging process, targeting various hallmarks of aging.

First, exercise may promote DNA repair and maintenance, enhancing genome stability and reducing the accumulation of DNA damage over time [192–195]. It activates DNA repair enzymes and increases the expression of proteins involved in DNA damage response pathways [196, 197]. Additionally, exercise can modulate telomere length and telomerase activity, which are associated with cellular senescence and aging [198–205].

Second, exercise mitigates oxidative stress [36, 45, 206], a key contributor to cellular aging and the pathogenesis of age-related diseases in various organ systems [42, 82, 84, 207–221]. It enhances antioxidant defenses, increases the expression of endogenous antioxidant enzymes, improves mitochondrial function, and reduces the production of reactive oxygen species (ROS) [222–224].

Third, exercise influences cellular senescence and inflammation, two interconnected hallmarks of aging. Cellular senescence refers to a state of irreversible cell cycle arrest that occurs in response to various stressors, such as DNA damage. Senescent cells accumulate with age and are implicated in tissue dysfunction and the pathogenesis of age-related diseases [80, 225–232]. Cellular senescence is characterized by the secretion of a complex mixture of pro-inflammatory molecules, known as the senescence-associated secretory phenotype (SASP) [233]. The SASP includes a variety of inflammatory cytokines, chemokines, growth factors, and enzymes involved in remodeling of the extracellular matrix (i.e., matrix metalloproteinases), which can promote local and systemic inflammation. This chronic low-grade sterile inflammation, often referred to as “inflammaging,” contributes to the development of a wide range of age-related diseases and tissue dysfunction. Exercise has been shown to attenuate the accumulation of senescent cells [234, 235] and reduce the production of pro-inflammatory cytokines [236–239], thereby promoting an anti-inflammatory environment and dampening inflammaging.

Fourth, exercise modulates metabolism and energy homeostasis, regulating key nutrient sensing pathways such as insulin/IGF-1 signaling, the mammalian target of rapamycin (mTOR) pathway [240–244], and activating sirtuins and AMP-activated protein kinase (AMPK) [245–249]. These pathways play a crucial role in the regulation of aging by sensing the availability of nutrients and energy levels in cells and modulating various cellular processes including metabolism, mitochondrial function, protein synthesis, and stress responses and cellular resilience.

Fifth, exercise also exerts beneficial effects on the mitochondria. Members of the sirtuin family of NAD^+^-dependent deacylases (SIRT-1, SIRT-3) play a critical role in regulation of mitochondrial biogenesis and bioenergetics, cellular resilience, and organismal lifespan [83, 219, 250–263]. Aging is associated with a decline in mitochondrial function, partially driven by mitochondrial DNA damage, dysregulation of mitochondrial gene expression, decline in SIRT-1 and SIRT-3 activity, and uncoupling of the electron transport chain [264–270]. This reduction in mitochondrial function can promote various age-related conditions, including sarcopenia and cardiovascular and cerebrovascular diseases [84, 207, 212, 264, 269, 271–273], due to increased mitochondria-derived production of reactive oxygen species (ROS), impaired cellular energetics, decreased cellular adenosine triphosphate levels, increased apoptosis, and cellular injury. Importantly, exercise has been shown to counteract these processes and improve mitochondrial function [196, 222, 223, 274–276]. Protective mechanisms induced by physical activity include the activation of the PGC-1α-dependent pathway, which promotes mitochondrial biogenesis, the reduction of mitochondrial ROS production and activation of autophagy, and the mitochondrial unfolded protein response [222, 223, 274–283]. Aerobic training sessions have been shown to upregulate sirtuins in skeletal muscle and other tissues, which in turn activates biogenesis and mitochondrial oxidative capacity [248, 262, 284–290]. Additionally, studies have demonstrated that resistance training can increase the activity of complex IV enzymes, which is associated with improved oxidative capacity. These findings have been observed in both animal and human studies, indicating the positive impact of exercise on mitochondrial function. While in-depth studies investigating the effects of walking on mitochondrial function are limited, initial studies have shown promising effects of walking interventions on mitochondrial function [291]. Future studies should determine how regular walking affect mitochondrial biogenesis and mitochondrial function in older adults in different tissues. Importantly, the quality of mitochondrial function can also influence an individual’s ability to engage in physical activity. Thus, there is a reciprocal relationship between physical activity and mitochondrial function, reinforcing the importance of exercise as a means to promote healthy aging and maintain optimal cellular metabolism.

Sixth, exercise affects cellular and tissue regeneration, promoting the maintenance and functionality of stem cells [292, 293]. It stimulates the release of growth factors and cytokines that support tissue repair and regeneration [145, 294–298].

Finally, exercise exerts a significant influence on the epigenetic regulation of aging processes, encompassing DNA methylation [299, 300], sirtuin activation, and histone acetylation [285, 287], thereby modulating gene expression patterns associated with aging and contributing to the maintenance of a youthful cellular phenotype. Various aging clocks have been developed to estimate biological age by measuring specific molecular and cellular biomarkers, including DNA methylation patterns and gene expression profiles. Exercise has emerged as a promising modality to positively influence biological age [300]. Studies have demonstrated that regular physical activity, including both aerobic and resistance exercise, is associated with a slower rate of biological aging as measured by different aging clocks. However, further research is needed to fully understand the extent to which exercise in general and walking interventions in particular can influence and reverse biological aging and to explore the potential of exercise interventions as a means to target and modify the trajectory of biological age.

By targeting these evolutionarily conserved mechanisms of aging, exercise exerts a holistic and profound impact on the aging process, promoting enhanced cellular function, tissue health, and overall longevity. Exercise paradigms serve as powerful interventions capable of delaying cellular aging processes, postponing the onset of age-related diseases and fostering healthy aging, as supported by a wealth of evidence from both preclinical and clinical studies. Future research is warranted to elucidate the extent to which uncomplicated, self-directed walking interventions can confer comparable benefits [237, 238, 301–303], as this knowledge holds great potential for promoting accessible and effective strategies for healthy aging.

### Adverse effects of walking

Despite the substantial health benefits associated with regular physical activity, vigorous-intensity physical activity may act as a trigger for cardiovascular outcomes such as ventricular arrhythmias, sudden cardiac arrest, sudden cardiac death, and acute coronary syndromes such as myocardial ischemia and myocardial infarction, transient ischemic attacks (TIAs), and cerebrovascular accidents strokes [13]. The risk of these outcomes is greatest in athletes and in people who do not habitually perform such intense physical activity. In athletes, it appears the intensities and volumes of these vigorous-intensity physical activity regimens far exceed those proposed by guideline recommendations [8, 9, 304]. Nevertheless, there is unequivocal evidence that the benefits of physical activity outweigh its potential adverse effects in healthy individuals. Walking is described as a low- to moderate-intensity physical activity; there is currently little evidence to suggest an increase in injuries or serious adverse events due to walking apart from a few isolated reports of calf injuries and falls, which occurred in people with conditions that put them at risk of these events [28]. However, too much walking could also be harmful especially in individuals who are not properly conditioned. If new to walking, it is essential to start slowly and gradually build up your duration and intensity.

## Optimizing physical activity: from brisk walking to step goals and health benefits

There is irrefutable evidence that adherence to current physical activity guideline recommendations [8, 9, 19] can reduce the risk of chronic diseases such as CVD and T2D and contribute to overall health. Brisk walking is an example of a moderate-intensity physical activity that counts towards the weekly recommended physical activity goals. While any type of walking can be beneficial, walking at a faster pace is associated with better cardiovascular and overall health compared to walking at a slow pace. A brisk walk of at least 30 min per day for 5 days allows one to meet the current physical activity guideline recommendations of at least 150–300 min of moderate-intensity aerobic physical activity per week [8, 9, 19]. Emerging data from prospective studies suggest that the cardiovascular and mortality benefits of physical activity can be achieved through both concentrated and spread-out patterns of activity [305]. The so-called weekend warrior pattern, with physical activity concentrated in 1 or 2 sessions per week, may be suitable for individuals with busy lifestyles who cannot meet the recommended physical activity levels. However, it should be noted that some beneficial effects of physical activity, such as reductions in blood pressure and lipids, are acute and need to be sustained by chronic regular physical activity [306, 307]. Additionally, the weekend warrior pattern may be more likely to be associated with musculoskeletal injuries and may not be suitable for people with comorbidities or musculoskeletal disorders.

It has been proposed that physical activity recommendations should be translated into step- or pedometer-based guidelines, as this could increase the clinical and public health impact of physical activity promotion [69, 76, 308]. A recent review of objectively measured physical activity types with clinical outcomes demonstrated that step count was the strongest and most consistently associated with a wide range of clinical outcomes [309]. In a study that sought to convert physical activity recommendations into a pedometer-based step goal, moderate-intensity walking was estimated to be approximately equal to at least 100 steps/min [308]. To achieve physical activity recommendations of at least 150 min per week of moderate-intensity physical activity, individuals needed to achieve the goal of walking a minimum of 3000 steps in 30 min for 5 days per week [308]. A goal of 10,000 steps per day has been widely promoted for decades as being the number associated with optimal health benefits [89, 310]. Recent evidence suggests that aiming for 8000 to 10,000 steps per day can substantially reduce the risk of CVD, diabetes, dementia, and premature death, and this goal is more attainable than the widely promoted reference of 10,000 steps per day [24, 69, 76, 86, 89]. Research suggests that the relationship between step count and health outcomes follows a curvilinear pattern, indicating that the benefits associated with increasing step count may be more pronounced for individuals with lower step volumes [76]. As step count increases, the protective effects on health outcomes tend to attenuate [76]. However, it is important to note that these recommendations may differ for aging adults. Specifically, a study has shown that even a modest increase in step count can have a significant impact on all-cause mortality in older adults aged 60 or more. In this study, it was found that taking as few as 6000 steps per day was associated with a reduction in mortality risk [24]. This highlights the importance of encouraging regular physical activity, such as walking, among aging individuals to improve their overall health and longevity. Another aspect to consider in relation to step count is cadence, which refers to the number of steps taken per minute. A study conducted in elderly patients found that those with a cadence of 100 steps or more per minute had a 21% lower risk of all-cause mortality compared to individuals with slower cadences [311]. Furthermore, for each ten-step increase in cadence, there was an additional 4% reduction in mortality risk. This suggests that not only the overall step count but also the pace or cadence at which one walks may have implications for health outcomes, particularly in older adults. These findings highlight the importance of considering both step count and cadence in promoting physical activity among individuals of different age groups. Encouraging individuals to increase their step count, especially those with lower step volumes, can have significant health benefits. Additionally, emphasizing the importance of maintaining a brisk walking pace or higher cadence may further enhance the positive impact on health outcomes, particularly in the older adult population.

Although the dose-response relationships between walking and cardiovascular risk factors have not been well quantified, pooled analysis of RCTs that have evaluated walking interventions for a minimum of 4 weeks has reported clinically important reductions in SBP and DBP of approximately 4–5 and 2 mmHg, respectively [25, 29, 32]. These blood pressure reduction effects are more pronounced in adults with high baseline blood pressure [49]. A 2-mmHg reduction in SBP could reduce mortality from stroke and vascular causes by 10% and 7%, respectively [312]; SBP reductions of 5–7 mmHg among individuals with hypertension translate to a 20–30% reduced risk of CVD [313], and a 2-mmHg reduction in DBP could reduce the risk of CHD by 6% and stroke and TIAs by 15% [314].

Additionally, walking has been demonstrated to increase levels of CRF [16–18], a strong predictor of adverse cardiovascular outcomes [315, 316], and middle-aged individuals who meet current recommendations for moderate-intensity physical activity (such as walking) are more likely to achieve at least moderate levels of CRF [317, 318].

## Conclusion and perspectives

In conclusion, the evidence overwhelmingly supports walking as a powerful anti-aging intervention that can reduce the risk of chronic age-related diseases such as CVD, hypertension, T2D, and cancer. Walking also improves pain and function in musculoskeletal disorders, promotes sleep and mental health and increases resilience. A brisk walk for at least 30 min, 5 days a week, is recommended to meet physical activity guidelines. Emerging data suggest that both concentrated and spread-out physical activity patterns can provide similar cardiovascular and mortality benefits. Step count is a strong and consistent predictor of clinical outcomes, and aiming for 8000 to 10,000 steps per day could substantially reduce the risk of a range of age-related diseases. Although some benefits of physical activity are acute, sustained and regular physical activity is necessary to maintain these effects. Overall, walking is a simple and effective intervention that can be easily integrated into daily routines to promote healthy aging and prevent chronic age-related diseases. Although it is not as high intensity as other physical activity types such as running, its health benefits are substantial and are irrespective of age, sex, race, or geographical location. Incorporating regular walking into daily routines should be encouraged as a key strategy for healthy aging and disease prevention.

Despite established physical activity guidelines and targets in most countries, and the World Health Organization’s recommendation that all nations implement policies to facilitate physical activity regardless of age or disability, global participation in physical activity has not improved over the last two decades. Recent estimates indicate that one in four adults do not meet aerobic exercise recommendations [319].

Walking-based interventions have the potential to improve health outcomes and promote healthy aging in a variety of populations, including employees at sedentary jobs at the workplace, older adults, individuals with chronic conditions, and those at risk for age-related diseases. One key advantage of walking-based interventions is their accessibility and affordability. Walking requires no special equipment or facilities and can be done at any time of day, making it an ideal form of physical activity for people of all ages and abilities. Furthermore, walking can be incorporated into daily routines, such as commuting to work, running errands, or taking leisurely strolls, making it an easy and convenient way to increase physical activity levels. Walking-based interventions have been shown to be effective in a variety of settings, including workplace health promotion programs [320], community-based programs [321], and clinical settings [322]. In particular, workplace walking interventions have been associated with improved productivity [323], reduced absenteeism [324], increased organizational commitment [320], improved job motivation [320], and lowered healthcare costs [325], while clinical walking interventions have been shown to improve functional status [326], reduce falls [327], and enhance quality of life [326, 328] in individuals with chronic conditions.

Substantial inequalities in physical activity participation persist across demographic factors such as age, sex, disability, socioeconomic status, and geographic location [329, 330]. These data underscore the urgent need for tailored walking-based interventions that effectively address the root causes of these disparities to maximize the potential of physical activity to improve health outcomes.

There is an urgent need to invest in services and interventions that promote walking across all populations. Promising target populations include sedentary, less active, and obese individuals who are unable to engage in vigorous-intensity physical activity, those who do not have access to exercise facilities, and individuals who are just not aware of the health benefits of walking. Interventions, supports, and programs that have been documented to promote and increase walking include outdoor walking groups [28], community-based walking programs, use of pedometers [331], computer- or mobile phone-based interventions [332, 333], transportation walking [334], and school and workplace initiatives [332]. Physicians specialized in preventive medicine, lifestyle medicine, or longevity medicine and health professionals have a key role to play in prescribing walking to their patients, especially those individuals who are unable to engage in vigorous-intensity physical activities.

To advance the field of geroscience, preventive medicine, and public health, future research should prioritize several areas of inquiry. First, research should quantify the frequency, duration, intensity, and volume of walking required to improve risk factors for CVD and other age-related diseases. Second, there should be a focus on describing the dose-response relationships between walking and various health outcomes, including the identification of thresholds for optimal benefit. Third, it is important to identify and evaluate other strategies for promoting and sustaining participation in walking over the long term. Finally, physical activity guideline recommendations based on step-counts for various populations, including different occupational groups, need to be developed. Such research will provide valuable insights into the role of walking as an effective intervention for promoting healthy aging and preventing chronic age-associated diseases. Studies investigating the age-specific effects of exercise and walking on health outcomes are also warranted [335]. As older adults experience age-related declines in immune function, they are at increased risk of severe illness and death from infectious diseases. As the COVID-19 pandemic swept the globe, older adults were identified as a particularly vulnerable population due to their increased risk for severe illness and death from the SARS-CoV-2 virus [179, 180, 336–348]. As a result, there has been growing interest in developing interventions to boost the immune function of older adults and improve their overall health and resilience [349, 350]. Walking-based interventions and exercise programs have been identified as a promising approach for contributing to these goals [351–355]. Future research should continue to explore the potential of walking as a low-cost and accessible intervention for improving immune function and other health outcomes in older adults. Comprehensive healthy aging programs containing walking-based interventions are important for improving societal resilience to future pandemics and promoting healthy aging for all.
